# Deregulated expression and activity of Farnesyl Diphosphate Synthase (FDPS) in Glioblastoma

**DOI:** 10.1038/s41598-017-14495-6

**Published:** 2017-10-26

**Authors:** Mario Abate, Chiara Laezza, Simona Pisanti, Giovanni Torelli, Vincenzo Seneca, Giuseppe Catapano, Francesco Montella, Roberta Ranieri, Maria Notarnicola, Patrizia Gazzerro, Maurizio Bifulco, Elena Ciaglia

**Affiliations:** 1 Department of Medicine, Surgery and Dentistry “Scuola Medica Salernitana”, University of Salerno, Via Salvatore Allende, 84081 Baronissi Salerno, Italy; 2 Institute of Endocrinology and Experimental Oncology, IEOS CNR, Via Pansini 5, 80131 Naples, Italy; 3 Department of Molecular Medicine and Medical Biotechnology, University of Naples “Federico II”, Via Pansini, 80131 Naples, Italy; 4 Neurosurgery Unit A.O. San Giovanni di Dio e Ruggi d’ Aragona - Salerno’s School of Medicine Largo Città di Ippocrate, 84131 Salerno, Italy; 5 “G.Rummo” Medical Hospital, Department of Neurosurgery, Benevento, Italy; 6 National Institute of Gastroenterology “S. de Bellis”, Research Hospital, Castellana Grotte, Bari, 70013 Italy; 7 Department of Pharmacy, University of Salerno, Via Giovanni Paolo II 132, 84084 Fisciano Salerno, Italy

**Keywords:** CNS cancer, Lipid signalling

## Abstract

Glioblastoma (GBM), the most aggressive brain cancer, is highly dependent on the mevalonate (MVA) pathway for the synthesis of lipid moieties critical for cell proliferation but the function and regulation of key intermediate enzymes like farnesyl-diphosphate synthase (FDPS), up to now, remained unknown. A deregulated expression and activity of FDPS was the central research idea of the present study. FDPS mRNA, protein and enzyme activity were analyzed in a cohort of stage III-IV glioma patients (N = 49) and primary derived cells. FDPS silencing helped to clarify its function in the maintenance of malignant phenotype. Interestingly, compared to tumor-free peripheral (TFB) brain and normal human astrocytes (NHA), FDPS protein expression and enzyme activity were detected at high degree in tumor mass where a correlation with canonical oncogenic signaling pathways such as STAT3, ERK and AKT was also documented. Further, FDPS knockdown in U87 and GBM primary cells but not in NHA, enhanced apoptosis. With the effort to develop a more refined map of the connectivity between signal transduction pathways and metabolic networks in cancer FDPS as a new candidate metabolic oncogene in glioblastoma, might suggest to further target MVA pathway as valid therapeutic tool.

## Introduction

The control of cellular metabolism is essential for a normal cell behavior, and the role that aberrant cellular metabolism has in cancer is becoming increasingly evident. Indeed, a clear interplay between cancer cell signaling, cholesterol and lipid metabolism is coming to the light^1^. In this context, glioblastoma (GBM), the most common and lethal tumor of the central nervous system, is highly dependent on the mevalonate (MVA) pathway for survival^2^ and an abnormally active de novo synthesis of cholesterol from acetate and MVA in malignant glial cells, compared with their normal counterparts, has been documented^3^. For this, glioma cells, but not normal astrocytes, were sensitive to shutting down cholesterol synthesis through pharmacological inhibition of lanosterol synthase or CYP51A1^4^. Further, upregulation of the mevalonate and cholesterol synthesis pathways has been associated with poor patient survival in GBM^4^. Thus, looking for novel, effective therapeutic strategies for GBM with minimal side effects on healthy tissues, this pathway has become an important promising target. Intermediate products of the isoprenoid pathway, other than mevalonate, include farnesyl and geranylgeranyl residues, which are involved in the post-translational modification of intracellular signaling proteins critical to tumor growth and maintenance of the malignant phenotype, such as Ras and Rho GTPase^5^. This has drawn significant attention to inhibitors of protein prenyl transferases and to the off label use of statins, inhibitors of 3-hydroxy-3-methyl- glutaryl-CoA (HMG-CoA) reductase (HMGCR), the rate-limiting enzyme in the mevalonate pathway. Indeed, multiple *in vitro* and *in vivo* studies have shown that statins have a wide range of anticancer activities in various cancers^6^. In brain, pitavastatin and cerivastatin at the same doses as those used to control hypercholesterolaemia, were an effective approach to inhibit GBM tumor growth and proliferation *in vitro* and in U87MG xenograft mouse *in vivo*^7^. A decreased cell migration in response to treatment with atorvastatin, probably due to inhibition of matrix metalloproteinase 2 (MMP2), was also described^8^. A significant anti-tumor activity has been reported with concurrent lonafarnib, an oral farnesyl transferase inhibitor, radiation, and temozolomide, in a murine model of glioblastoma^9^. However, in addition to the enzymes that are targeted in current preclinical and clinical applications, other key component of MVA pathway, not extensively studied to date, might represent potential druggable targets for cancer therapy. In particular, Farnesyl Diphosphate Synthase (FDPS) is a branch point enzyme in the synthesis of sterols and isoprenylated cellular metabolites. Mainly known to mediate immunoregolatory functions^10,11^ its activity and expression have been also documented in human colon cancer^12^ and certain other neoplastic disorders. Indeed high mRNA levels of FDPS together with those of additional isoprenoid pathway gene transcripts have been correlated with poor patient prognosis and reduced survival in a meta-analysis of six microarray datasets of primary breast cancers^4,13^. An augmented FDPS expression was also found in K-ras and H-ras transformed FRTL5 thyroid cells^14^ and FDPS, in cooperation with H-ras oncogene, displayed neoplastic transformation potential in primary rat embryo fibroblast cells^15^. As recently found in U343MG glioma cells, the notable tumor suppressor p53 was able to induce the expression of a group of enzymes of the MVA pathway including HMGCoA reductase, MVA kinase, FDPS and farnesyl diphosphate farnesyl transferase 1^3^. Further, in human U87MG glioma cell line, FDPS played an important role in attenuating paclitaxel-induced cell death by affecting p53 and c-Jun N-terminal kinase (JNK)^16^. Overall, these findings prompted us to better underpin the contribution of FDPS to malignant gliomas.

## Material and Methods

### Reagents and Abs

Epidermal growth factor (EGF) was diluted in a buffer containing a stabilizer (5% Trehalose) and added to cell cultures at the indicated concentration. Human EGF was purchased from Peprotech (London, UK). In transfection procedure Lipofectamine 3000 were from Invitrogen. Optimem were from Gibco (#51985). For western blot analysis the following antibodies were used: rabbit polyclonal anti-human FDPS (#ab38854) at 1:1000 and rabbit polyclonal anti-human β-actin (#ab16039) at 1:1000 were purchased from Abcam (Cambridge, UK), mouse monoclonal anti-human α-Tubulin (#T5168) at 1:4000 from Sigma-Aldrich Inc. (St Luis, MO, USA), mouse monoclonal anti-human PCNA (#2586) at 1:2000, rabbit monoclonal anti-human Mcl-1 (#94296) at 1:1000, rabbit monoclonal anti-human BCL-XL (#2764) at 1:1000, rabbit polyclonal anti-human phospho-STAT3 (p-STAT3; Tyr705) (#9145) at 1:2000, rabbit polyclonal anti-human STAT3 (#4904) at 1:2000, rabbit monoclonal anti-human Phospho-p44/42 MAPK (p-Erk1/2; Thr202/Tyr204) (#4370) at 1:2000, rabbit monoclonal anti-human p44/42 MAPK (#4695) at 1:1000, rabbit monoclonal anti-human Phospho-Akt (p-AKT; Ser473) (#4060) at 1:2000 and rabbit monoclonal anti-human AKT (#4691) at 1:1000 were purchased from Cell Signaling Technology (Danvers, MA). Secondary HRP-linked goat anti-mouse or goat anti-rabbit IgG, were also purchased from Cell Signaling Technology (Danvers, MA).

### Cells and clinical samples

Normal Human Astrocytes (NHA) are normal human cells derived from human brain tissue and were cultured in recommended medium AGM™ BulletKit™ (Lonza). The human glioma cell lines U343MG (U343), U87MG (U87), U251MG (U251) and T98G (T98) were obtained from CLS Cell Lines Service GmbH (Eppelheim, Germany) or were kindly provided by Dr. Daniela Parolaro (University of Insubria, Italy). Small pieces of brain tissue containing tumor were collected at the time of craniotomy for tumor resection at the Neurosurgery Service of “G. Rummo” Medical Hospital (Benevento, Italy) and of “San Giovanni di Dio Ruggi d’Aragona” Medical Hospital (Salerno, Italy) and handled for further analysis according to the procedure previously established^17^ The tumors were diagnosed as astrocytoma (WHO grade I-III; n = 5), glioma (WHO grade II, n = 5) or glioblastoma multiforme (WHO grade IV; n = 39). There were not significant differences by gender, or age between the different groups (Supplementary Table 1). All tissue samples were collected in accordance with the ethical standards of the Institutional Committee of “G. Rummo” Medical Hospital (Benevento, Italy) and the experimental protocol was approved by “Campania Nord Ethical Committee” of “S. Giuseppe Moscati” Medical Hospital (Avellino, Italy) (DEL. N8548 04/20/2007, April 3, 2013 and DEL. 26/10/2016). The patients had been informed about the establishment of cellular models from their tumour and had given informed consent in written form.

The preparation of adherent primary cultures of brain tumor cells (designated as GBMn) was conducted accordingly to the procedure previously described by our group^17^.

Human glioma cell lines were cultured in EMEM (Lonza) supplemented with 10% heat-inactivated fetal bovine serum (Euroclone), 1% L-glutamine, 1% antibiotic mixture, 1% sodium pyruvate, 1% non-essential aminoacids (Euroclone).

### Apoptosis analysis

The evaluation of the apoptosis of U87MG glioma cell lines, NHA and patient-derived primary cell line (GBM39) was conducted by anti-human Annexin V (BioLegend, San Diego, CA, USA) and PI staining. Briefly, cells grown in 100-mm dishes for 48 h in EMEM, ABM or DMEMF12 supplemented were harvested with trypsin and washed in PBS. The cells were resuspended in Annexin V binding buffer (10 mM HEPES/NaOH, pH 7; 140 mM NaCl; and 2.5 mM CaCl2) and stained with Annexin V-FITC for 20 min at room temperature (RT) and then with PI at RT for additional 15 min in the dark. The cells were acquired by flow cytometer within 1 h after staining. At least 10,000 events were collected, and the data were analyzed by CellQuest Pro software (Becton Dickinson, San Jose, CA). Data are expressed as logarithmic values of fluorescence intensity.

### Short interfering RNA transfection

Control or target-specific siRNAs were purchased from Sigma and transfected at a concentration of 20 nmol/L using a Lipofectamine RNAi MAX kit (Invitrogen) according to the manufacturer’s instructions. Knock-down level of target genes was determined using qRT-PCR. FDPS siRNA transfection was carried out according to manufacturer’s instructions. First, siRNA FDPS (Ambion) and negative control siRNA (Silencer Negative Control, Ambion) were dissolved in Opti-MEM serum-free media. Both FDPS and scrambled siRNA were delivered into the U87 cell cultures, NHA and patient-derived primary cell line (GBM39) plated 18 hours prior (approximately 80% confluency) via Lipofectamine RNAi MAX. The final concentration of FDPS and scrambled siRNA in culture was 100 nM. The cells were incubated with the transfection reagents for 48 hours. Cell media was replaced with serum-free media to induce starvation and was added EGF (final concentration 50ng/mL) for 8 minutes for U87 and GBM39 cells. The cells were then harvested for analysis of protein knockdown via Western Blot and for the determination of cell cycle arrest and the apoptosis induction, as reported above.

### Real-time PCR

Quantitative RT-PCR RNA extraction and quantitative reverse transcription-PCR (qRT-PCR) were performed as previously described^3^. Gapdh was used as the housekeeping gene expression control. Total RNA was isolated from 5 × 10^6^ cells using TRIzol® reagent (Invitrogen, Paisley, UK), according to the manufacturer’s instructions. Complementary DNA (cDNA) was transcribed using SuperScript II Reverse Transcriptase (Invitrogen, Paisley, UK), starting from 1 μg/μl of high pure RNA and samples were tested in triplicate using the SsoFast EvaGreen reagents (Bio-Rad). qRT-PCR protocol was: pre-heating step for 3 minutes at 95 °C, then 40 cycles at 95 °C for 10 seconds and 60° for 30 seconds and last end-step at 65 °C for 10 seconds. Finally, results were analyzed with 2^−ΔΔCt^ method.

### Western blot (WB) analysis

For analysis of protein levels cells were grown in p60 tissue culture plates at a density of 2 × 10^4^ cells/cm^2^ for 24 h, were washed with PBS, harvested and lysed in ice-cold RIPA lysis buffer (50 mM Tris-HCl, 150 mM NaCl, 0.5% Triton X-100, 0.5% deoxycholic acid, 10 mg/ml leupeptin, 2 mM phenylmethylsulfonyl fluoride, and 10 mg/ml aprotinin). Tumor pieces were disrupted for protein extraction by gentle homogenization (Potter-Elvehjem Pestle) in cold RIPA buffer. After removal of cell debris by centrifugation (14,500 g for 20 min at 4 °C), the proteins were estimated. About 30 µg of proteins was loaded on 10, 12 or 15% SDS–polyacrylamide gels under reducing conditions and then transferred to nitrocellulose membranes. The blots were blocked with 5% nonfat dry milk (Bio-Rad, Richmond, CA, USA) in Tris-buffered saline containing 0.1% Tween-20 (TBST) for 1 h at room temperature and incubated with the specific antibody. Immunodetection of specific proteins was carried out with horseradish peroxidase-conjugated donkey anti-mouse or anti-rabbit IgG (Biorad, Hercules, CA), using the enhanced chemiluminescence (ECL) system (Amersham Pharmacia Biotech, Piscataway, NJ) according to the manufacturer’s instructions and then exposed to X-ray films (Amersham Biosciences). Immunoreactive bands were quantified with Quantity One 1-D analysis software (Bio-Rad). To ascertain equal protein loading in Western blots of cell lysates, membranes were probed with an antibody raised to α-tubulin (Sigma-Aldrich, St. Louis, MO, USA) or β-actin (Abcam, Cambridge, UK).

### Cell cycle analysis

U87MG cells, after short interfering RNA transfection, were collected, fixed in 70% ethanol, and kept at −20 °C overnight. Propidium iodide (PI; 50 μg/ml) in PBS containing 100 U/ml DNase-free RNase was added to the cells for 15 min at room temperature. Cells were acquired by a FACS Calibur Flow Cytometer (BD Biosciences, San Jose, CA, USA). The analysis was performed with ModFit LT v3.2 (Verity Software House, Inc., Topsham, ME, USA); 10, 000 events, corrected for debris and aggregate populations, were collected.

### FDPS activity assay

Briefly, FPPs was assayed in 150 µl containing 25 mM Hepes, pH = 7, 2 mM MgCl2, 1 mM dithiothreitol, 5 mM KF, 1% n-octyl-ß-glycopyranoside, 3.3 µM [4-14 C] IPP (18 Ci/mmol), 3 µM unlabeled IPP and 20 µM geranyl diphosphate. Reactions were started by adding 40 µl of peroxisomal fraction containing 100 µg of total protein and incubated for 45 min at 37 °C. Reactions were stopped by the addition of 150 µl 2.5 N HCl in 80% ethanol containing 100 µg/ml farnesol as a carrier. The samples were hydrolyzed for 30 min at 37 °C to convert the FPP to farnesol and neutralized by the addition of 150 µl of 10% NaOH. The reaction product (farnesol) was extracted into 1 ml of n-hexane and an aliquot (200 µl) of the organic phase was used for radioactivity counting. One unit of enzyme activity is defined as the amount of enzyme required to synthesize one pmol of FPP per min. Parallel samples were assayed to evaluate the total and the nonspecific radioactivity. In all experiments, enzyme assays were carried out in duplicate. The coefficient percentages of intra- and interassay variation were 3 and 4%, respectively.

### Statistical analysis

Statistical analysis was performed in all the experiments shown by using the GraphPad prism 6.0 software for Windows (GraphPad software). For each type of assay or phenotypic analysis, data obtained from multiple experiments are calculated as mean ± SD and analyzed for statistical significance using the 2- tailed Student t-test, for independent groups, or ANOVA followed by Bonferroni correction for multiple comparisons. Correlation analyses were performed using the Pearson rank‐sum test. P values less than 0.05 were considered significant. *P < 0.05, **P < 0.01 and ***P < 0.001.

### Data Availability

The datasets generated during and/or analysed during the current study are available from the corresponding author on reasonable request.

## Results and Discussion

**Figure 1:**
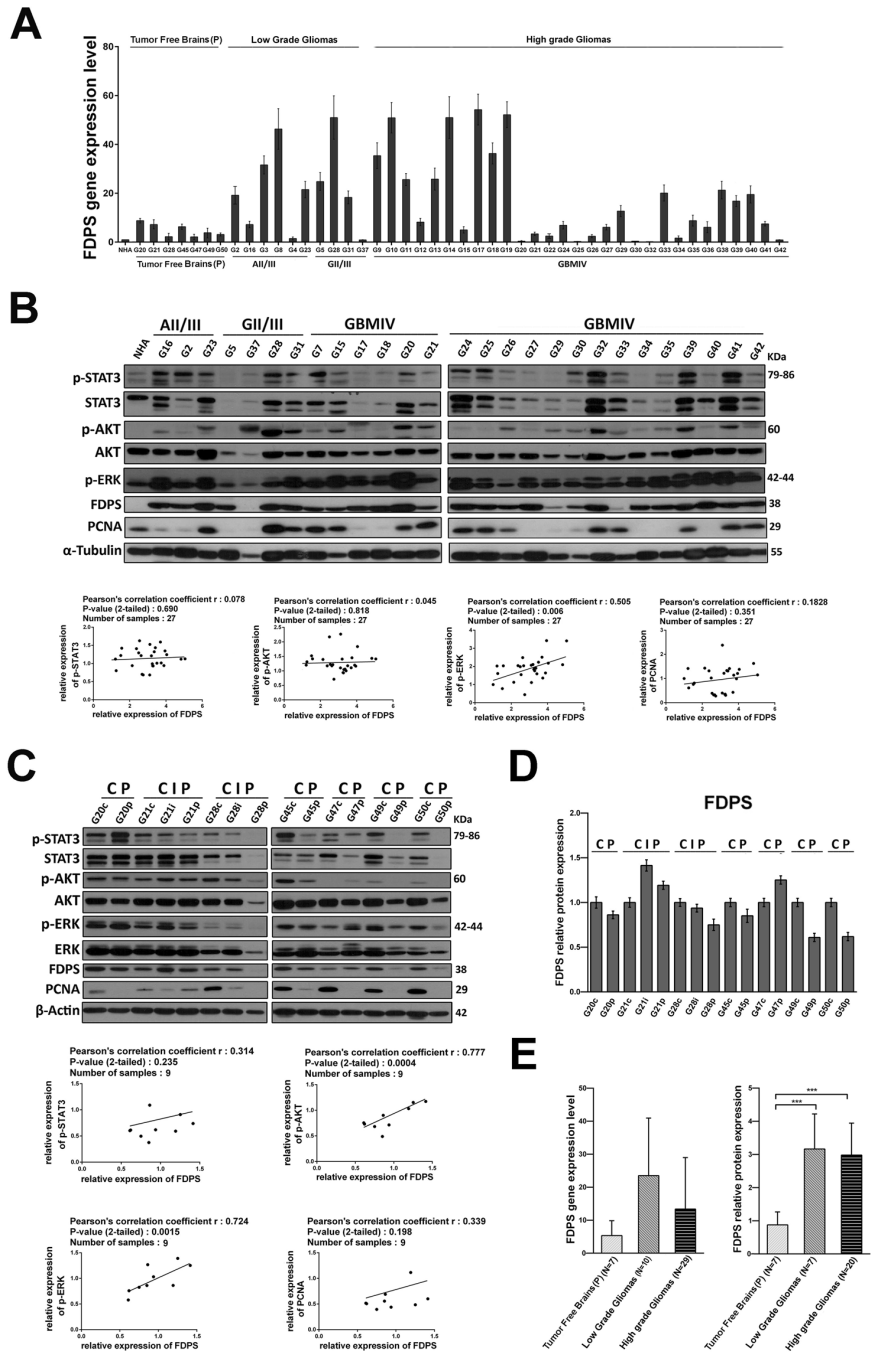
Dysregulated FDPS expression in GBMs. (**A**) Real-time PCR analysis of FDPS in NHA, 7 tumor free brain tissues and 39 glioma tissues (G2-G42). Data, expressed as fold change units, were normalized with β-actin and referred to the NHA considered as calibrator. Columns represent mean ± SD of the results performed in triplicates. (**B**) Representative Western blot showing the basal protein levels of p-STAT3, STAT3, p-AKT, AKT, p-ERK, FDPS and PCNA in NHA and 27 tumor brains (G16 astrocytoma grade II, G2-23 astrocytoma grade III, G5-G31 glioma grade III, G7-G42 glioblastoma grade IV); α-Tubulin was used as loading control. Each set of samples (NHA-G21) and (G24-G42) has been run in parallel. The two panels show the representative western blots of three different experiments performed with similar results whose densitometric analysis is showed in Supplementary Fig. 2. The graphs below represent correlation analysis of western blot results for the protein expression of FDPS and the protein expression of p-STAT3, p-AKT, p-ERK and PCNA. Plotted values are normalized on corresponding ones in NHA (**C**) Western blot analysis for p-STAT3, STAT3, p-AKT, AKT, p-ERK, FDPS and PCNA in 7 tumor brains divided into the Central (**C**), Intermediate (I), and Peripheral (P) fraction as explained in Supplementary Fig. 1. β-actin was used as loading control. Each set of samples (G20c-G28p) and (G45c-G50p) has been run in parallel. The two panels show representative Western blots of three different experiments performed with similar results whose densitometric analysis is showed in Supplementary Fig. 3. The graphs below represent correlation analysis of western blot results for the protein expression of FDPS and the protein expression of p-STAT3, p-AKT, p-ERK and PCNA. Plotted values are normalized on corresponding ones in tumor infiltrated Central tissue (**C**). (*D*) FDPS quantitative analyses of the western blot results of Fig. 1C. FDPS values in I and P fractions are normalized on corresponding ones in tumor infiltrated Central tissue. Histograms represent mean ± SD in densitometry units of scanned immunoblots from the 3 different experiments. (**E**) FDPS gene (*left panel*) and protein (*right panel*) expression in tumor free brains, low grade gliomas and high grade gliomas groups as respectively detected by RT-PCR and western blotting. Quantitative analyses of the results are shown in histograms. Data are presented as the mean ± SD of the results performed in triplicates. (ANOVA, ***p < 0.001).

Given the putative oncogenic role of an aberrant mevalonate pathway in glioblastoma, we looked for potential expression changes of the FDPS key intermediate enzyme in patients. First, we investigated the pattern of expression of FDPS in gliomas, by extracting total RNA and proteins from 49 primary glioma samples, among which there were grade II, grade III, and grade IV glioma tissues. Normal human astrocytes (NHA) and 7 peripheral tumor free brains, obtained through the experimental procedure described in Supplementary Fig. 1, were used as control. As therapy might be a confounding factor for further analyses, we selected only treatment-naïve patients, that is without prior radiation therapy and chemotherapy (Supplementary Table 1). Overall, eventhough without any apparent association with tumor type and grade (Fig. 1E), FDPS was found upregulated in terms both of mRNA and protein levels in glioma patients samples compared with NHA and peripheral tumor free brains (Fig. 1A,B). Overall a positive signal of FDPS was found in almost all glioma tissues, whereas an almost undetectable level of FDPS was observed in NHA, cell basis for comparison for all subsequent analysis. Specifically as indicated in the comparative histograms of both protein and mRNA levels of FDPS in the three groups (Fig. 1E), the relative protein expression of FDPS in high grade gliomas was significantly higher than that in peripheral tumor free brains (2.98 ± 0.97 vs. 0.87 ± 0.39; n = 20 vs. 7; P < 0.001) (Fig. 1E, right). Referred to the NHA considered as calibrator, the FDPS mRNA levels in the high grade group was higher compared with those in peripheral tumor free brains (13.36 ± 15.61 vs. 5.32 ± 4.52; n = 20 vs. 7) (Fig. 1E). At the same way, the relative protein expression of FDPS in low grade gliomas was significantly higher than that in peripheral tumor free brains (3.16 ± 1.07 vs. 0.87 ± 0.39; n = 7 vs. 7; P < 0.001). Interestingly, the FDPS mRNA levels in the low grade group was even higher compared with those in peripheral tumor free brains (23.56 ± 17.43 vs. 5.32 ± 4.52; n = 10 vs. 7) eventhough it failed to reach statistical significance. Concerning this point, the analysis of those samples where both RNA and proteins were available, showed that the dinamic range of transcript levels was not always reflected at the protein expression level. Indeed this is not surprising as protein expression level can be regulated by multiple mechanisms concerning mRNA processing and stability, post-transcriptional and translation process as well as protein half live which may all account for the lack of protein-mRNA correlation. In our opinion, this might also explain the lack of statistical significance for mRNA levels among different groups.

In line with published literature^17–19^, phosphorylation and thus kinases activity and transcription factor allowing survival (AKT, STAT3) and proliferation (ERK1/2) of tumor cells were also frequently elevated in GBM panel where an interesting directionality between FDPS and these common oncogenic signaling pathways was found in the majority of tumor tissues analyzed (Fig. 1B,C). Specifically, a positive correlation between the expression of FDPS and those of ERK (Pearson’s correlation coefficient r = 0.50; n = 27; 2-tailed P < 0.006) and of Proliferating Cell Nuclear Antigen (PCNA) (Pearson’s correlation coefficient r = 0.18; n = 27) was observed in Fig. 1B. Further when we moved to characterize intra-patient protein expression, FDPS levels in peripheral tumor free margins (P) was lower compared to those of both adjacent intermediate regions (I) and central tissues (C) of the dissected tumors from 5/7 representative patients (Fig. 1C,D). Of note, in this data set correlation analysis not only confirmed the existence of a positive correlation between FDPS expression and activation of the ERK pathway (Pearson’s correlation coefficient r = 0.72; n = 9; 2-tailed P < 0.001) and PCNA proliferative marker accumulation (Pearson’s correlation coefficient r = 0.33; n = 9) but clearly highlighted a similar positive correlation between FDPS levels and that of STAT3 (Pearson’s correlation coefficient r = 0.31; n = 9) and significantly that of AKT (Pearson’s correlation coefficient r = 0.77; n = 9; 2-tailed P < 0.001) oncogenic pathways (Fig. 1C). So, accordingly to the aberrant role of mevalonate pathway in tumor growth and progression, for the first time here we show that GBMs frequently display extremely variable expression levels of the FDPS protein which abnormally accumulated in pathological settings.

**Figure 2:**
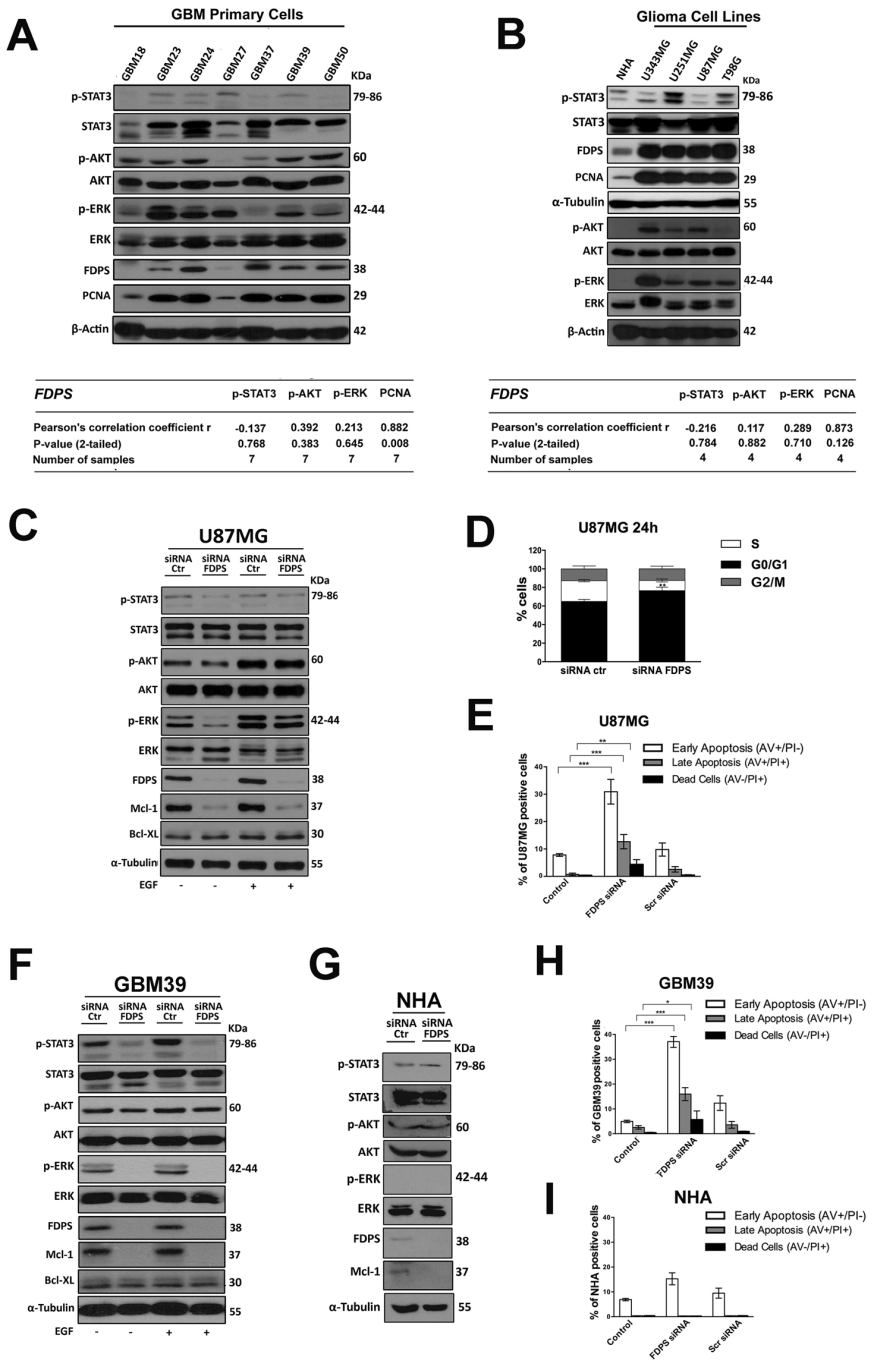
Functional analysis of the role of FDPS in GBMs. (**A**,**B**) Representative Western blot showing pSTAT3, STAT3, p-AKT, AKT, p-ERK, ERK FDPS and PCNA protein levels in 7 human primary glioma cell lines established from the indicated cancer patients (G18, G23, G24, G27, G37, G39, G50) (**A**), in NHA and in the four indicated glioma cell lines; β-actin or α-Tubulin were used as loading control. Panels show representative blots of three different experiments performed with similar results. The tables below report correlation analysis of western blot results for the protein expression of FDPS and the protein expression of p-STAT3, p-AKT, p-ERK and PCNA. (**C**) U87MG cells or U87MG cells transfected with siRNA FDPS were cultured for 48 h in presence or absence of EGF in the last 8 minutes before cells lysis; cell lysates were immunoblotted for p-STAT3, STAT3, p-AKT, AKT, p-ERK, ERK, FDPS, Mcl-1, BCL-XL and α-Tubulin as loading control. Data are representative of 3 independent experiments performed with similar results. (**D**) Distribution of U87MG cells or U87MG cells transfected with siRNA FDPS in the different cell cycle phases. All the results shown are representative of three independent experiments performed in duplicate, expressed as mean ± SD (ANOVA, **p < 0.01*vs* control). (**E**) Cytofluorimetric assessment of apoptosis in U87MG cells or U87MG cells transfected with siRNA FDPS or Scramble siRNA. Histograms indicate the total percentage of early (AV + /PI- cells) and late apoptotic events (AV + /PI + cells) as well as necrotic cells (AV-/PI + cells). All the results shown are representative of three independent experiments (ANOVA, *** p < 0.001, ** p < 0.01). (**F**) Patient-derived primary cell line (GBM39) or GBM39 transfected with siRNA FDPS were cultured for 48 h in presence or absence of EGF in the last 8 minutes before cells lysis; cell lysates were immunoblotted for p-STAT3, STAT3, p-AKT, AKT, p-ERK, ERK, FDPS, Mcl-1, BCL-XL and α-Tubulin as loading control. Data are representative of 3 independent experiments performed with similar results. (**G**) NHA cells or NHA cells transfected with siRNA FDPS were cultured for 48 h; cell lysates were immunoblotted for p-STAT3, STAT3, p-AKT, AKT, p-ERK, ERK, FDPS, Mcl-1 and α-Tubulin as loading control. Data are representative of 3 independent experiments performed with similar results. (**H**,**I**) Detection of apoptosis in patients-derived primary cell line (GBM39), both in basal condition and after FPDS silencing (**H**) and in NHA cells or NHA cells transfected with siRNA FDPS or Scramble siRNA (**I**). All the results shown are representative of three independent experiments (ANOVA, ***p < 0.001, **p < 0.01, *p < 0.05).

After confirming the lack of interference of serum-based culture conditions on FDPS protein levels (Supplementary Fig. 4), next we moved to compare FDPS protein expression in a panel of primary cell lines established from fresh resectioned tumoral masses of 7 selected individual patients with glioma. Here, the cells characterized by the highest FDPS levels (GBM24, GBM39, GBM50), with the exception of GBM37 derived from a low grade glioma, also expressed higher levels of the associated oncogenic signal transduction proteins than those derived from low to moderate FDPS levels tumors (GBM18, GBM27, GBM23) as suggested by the values of correlation analysis between FDPS expression and p-ERK (Pearson’s correlation coefficient r = 0.213; n = 7), PCNA (Pearson’s correlation coefficient r = 0.882; n = 7), and p-AKT (Pearson’s correlation coefficient r = 0.392; n = 7) expression in all GBM primary cells. As expected, a similar relationship between FDPS status and oncogenic potential was conserved in a panel of 4 glioma cell lines (U343, U251, U87, T98) and in normal astrocyte as their healthy counterpart. The results in Fig. 2B showed in fact, that protein expression of FDPS was up-regulated in all glioma cell lines tested compared with NHA and that with the exception of p-STAT3 (Pearson’s correlation coefficient r = −0.216; n = 4), it was positively correlated with p-ERK (Pearson’s correlation coefficient r = 0.289; n = 4), p-AKT (Pearson’s correlation coefficient r = 0.117; n = 4) and PCNA (Pearson’s correlation coefficient r = 0.873; n = 4) immunoreactivity.

Knockdown experiments were then performed to test the functional relevance of FDPS. As exemplified for U87 cell line, cells transfected with FDPS siRNA showed less active STAT3, AKT and ERK compared to cells transfected with scrambled siRNA (Fig. 2C), either when the signaling operated constitutively either when it was activated by EGF stimulus (50 ng/ml), eventhough to a less extent as clearly showed by the densitometric analysis in Supplementary Fig. 5. Of note, the decreased expression of the anti-apoptotic protein Mcl-1, which was regulated to transcriptional, post-transcriptional and post-translational level respectively by STAT3, AKT and ERK^20^ well fits with the inhibition of these cell signaling pathways by FDPS knockdown (Fig. 2C). The unchanged level of BCL-XL when cells were silenced, might suggest a key role for Mcl-1 in cell death sensitization of glioma cells by FDPS abrogation. This finding is consistent with the well known deregulated apoptotic pathways of cancer cells (vs normal ones), where an increased expression and stability of anti-apoptotic proteins Mcl-1 and Bcl-2 increases resistance to apoptosis^21^. Indeed as functional correlate, following cell cycle arrest in G0/G1 phase (Fig. 2D), FDPS knockdown associated to the induction of apoptosis, as the percentage of cells in early and late apoptosis were significantly higher in U87 cells transfected with FDPS (30,9 ± 4,5% and 12,6 ± 2,6%, p < 0,05) as compared to Scramble treatment (9,8 ± 2,4% and 2,5 ± 0,9%) (Fig. 2E), overall suggesting a putative role for FDPS in glioma cell growth and viability. In this context since Mcl-1 has also been implicated in resistance to a variety of commonly used chemotherapeutic agents^21^ we cannot exclude that FDPS might circumvent drug resistance. This is also true because the presence in FDPS promoter of sterol regulatory element (SRE) which usually serve to replenish MVA pathway metabolites following statins treatments^1^. Therefore targeting FDPS, by inhibiting these resistance feedback mechanisms may be useful to maximize the efficacy of treatments. Of note, primary GBM39 cells showed comparable responses to FDPS siRNA knockdown as observed in U87MG glioma cell line (Fig. 2F,H). This corroborated previous findings and allowed the discovery of novel pro-survival routes of the glioblastoma related to FDPS. Indeed the enzyme depletion by counteracting signal output from several oncogenic signaling pathways, possibly through the decrease of Ras isoprenylation^1^ or by the disruption of those lipid moieties which seem to be critical for the functionality and activity of epidermal growth factor receptor (EGFR), the most frequent oncogenic event occurring in GBM^18,19^, resulted in cell cycle arrest and then apoptosis of glioma cells. Interestingly, FDPS silencing has minimal impact on normal cells (NHA) (Fig. 2G) probably because this FDPS route has lower activity, as suggested by its barely detectable protein expression along that of Mcl-1 in both naive low passages NHA (Fig. 2B,G) as well as in NHA cultured for two weeks in different specific culture conditions (serum free MACS® Neuro Medium vs conventional 15% DMEM-F12) (Supplementary Fig. 4). These findings are consistent with those from Kambach *et al*.^4^ which also reported a similar downregulation of cholesterol biosynthesis enzymes in dense NHA for which they might be less susceptible to cytotoxicity of FDPS depletion than GBM cancer cell lines. Finally we cannot exclude that the failure in the NHA apoptosis induction by siRNA FDPS might be related to the inability of knockdown procedure to interfere with the STAT3 and AKT survival pathway in normal compartment.

**Figure 3:**
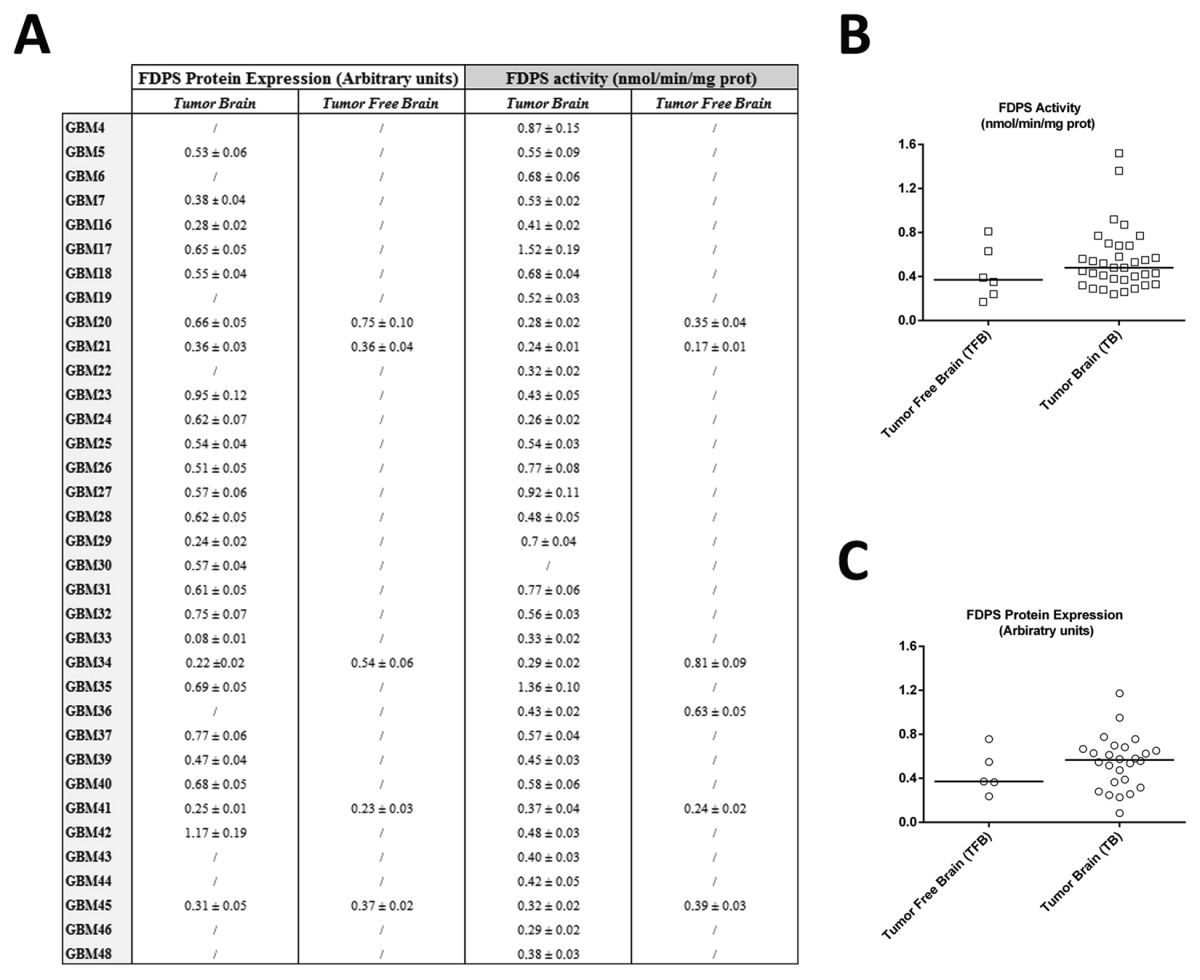
Densitometric analysis of FDPS protein expression and FDPS activity in tumor brain and in tumor free brain tissues. (**A**) Table of densitometric analysis values of FDPS protein expression normalized on own β-actin or α-Tubulin (arbitrary units) and FDPS activity values (expressed in nmol/min/mg prot) in G4-G48 glioblastoma tumor brain and in tumor free brain tissues; the same values were plotted in the next scatter plots (**B**,**C**) where bars indicate median expression. Results are representative of 3 different independent determination for each indicated patient brain tissues.

**Figure 4:**
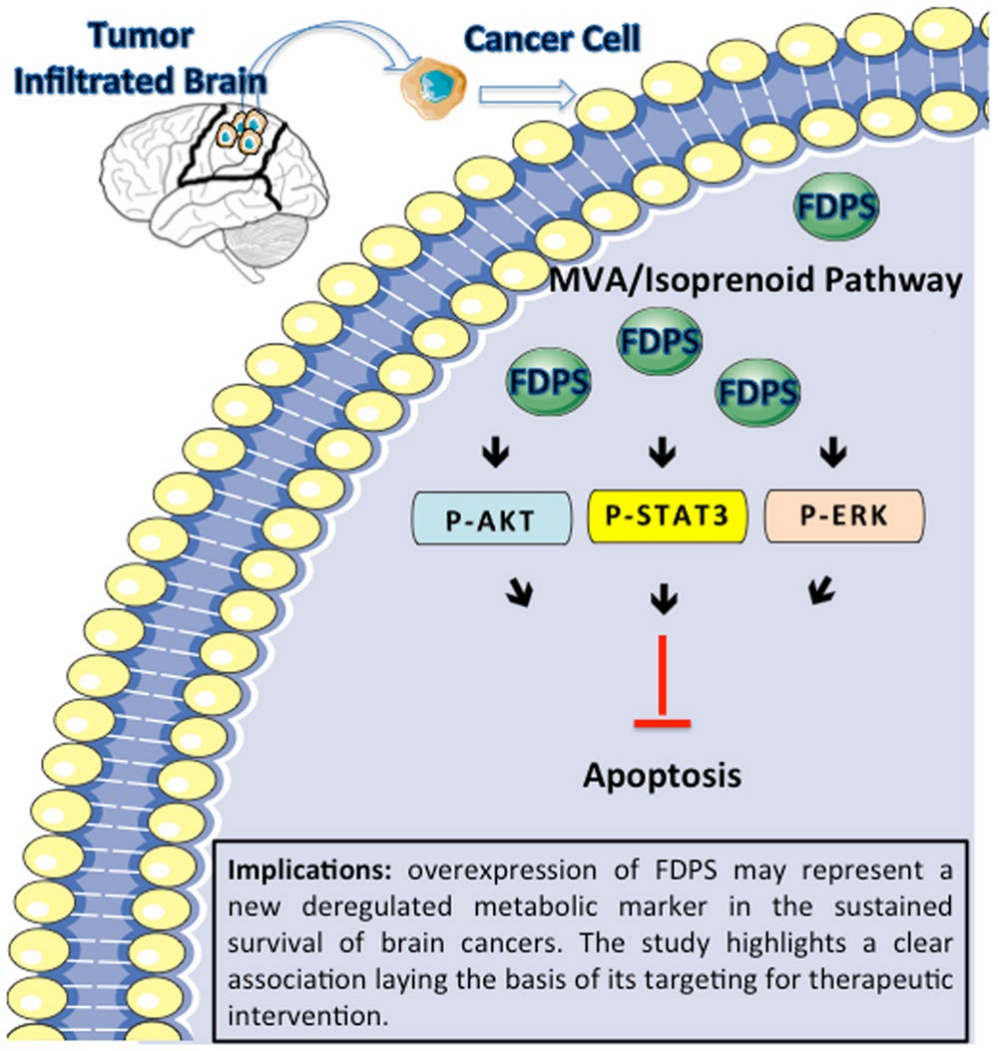
Schematic representation of the main molecular findings. Looking for novel brain cancer biomarkers, the protein, the enzymatic and the gene expression determinations of FDPS have been conducted in different intraoperative brain sample sites of a series of GBM patients. Compared with the normal human astrocyte (NHA) and Tumor Free Brains (TFB), human tumor infiltrated brains (TIB) displayed an aberrant expression of this enzyme (green). This led us to begin to shed light on the role of FDPS in cell survival mainly by modulating the oncogenic signalling pathways in GBM cancer cells.

To quantitatively prove FDPS enzyme functionality in the sustained survival of GBMs, its activity was measured by radiochemical assay. The enzymatic activity level of FDPS was determined in tumor infiltrated brain of 34 patients and in the normal surrounding tissues of 6 patients, for comparison. The differences in sample size between the two groups analysis are due to obvious ethical reasons. Surprisingly, FDPS activity was detectable in all brain tissues (Fig. 3) as a third parameter available to evaluate the intratumor FDPS levels. With regard to tumor free brain, eventhough not statistically significant, a trend increase in FDPS activity in GBM was found (0.48 *vs* 0.37 nmol/minmg total protein, median value) (Fig. 3B) and interestingly it followed that of FDPS protein levels for the same two different anatomical compartments (0.56 *vs* 0.36 arbitrary units, median value) (Fig. 3C), highlighting a functional relevance of FDPS overexpression. As FDPS is located within the mevalonate pathway before the bifurcation that leads to isoprenoid synthesis and cholesterol, it is conceivable that a fine modulation of isoprenoid levels might also follow that of FDPS in tissues and cell lines in a manner that need a much deeper analysis in the future. In the light of these findings, the documented increase in FDPS activity in tumor tissues might suggest not only its possible use as a reference marker for GBM diagnosis, but also its contribution to gliomagenesis, delineating this enzyme as a valid druggable target for therapy. Indeed this enzyme is already the pharmacological target of nitrogen-containing bisphosphonates (N-BP) which, in addition to being potent inhibitors of bone resorption, have been shown to exert *in vitro* and *in vivo* antitumor activity. In *in vitro* models of brain cancer, only few indirect evidence highlighted the efficacy of N-BP, mainly attributed to different biochemical mechanisms and without a clear relationship with FDPS status^22,23^. Here, we not only provide the rationale to their use but the intratumor FDPS activity can have clinical implication as its levels may be predictive of response to therapy, as for example the documented resistance to 5-Fluorouracil (5-FU) partly due to insufficient inhibition of thymidylate synthase (TS) activity in gastrointestinal cancer patients^24^ or the strong association between cisplatin-resistance and pyruvate kinase M2 (PKM2) overexpression and enzyme activity in advanced bladder cancer (BC)^25^. In conclusion, FDPS appears to be a new metabolic driver oncogene in GBM. Eventhough further analysis is required, in addition to follow-up studies, in order to confirm these observations, FDPS targeting, with statins, farnesyltransferase inhibitors (FTIs) and N-BP or new safe drugs, might represent a valid MVA flux control point in antiglioma pharmacological research (Fig. 4).

## Electronic supplementary material

Supplementary Information
