# Supplementary material for: Deregulated expression and activity of Farnesyl Diphosphate Synthase (FDPS) in Glioblastoma
Source: Sci Rep. 2017 Oct 26;7:14123. doi: 10.1038/s41598-017-14495-6 (PMC5658376; doi:10.1038/s41598-017-14495-6)

Supplementary informations

for

Deregulated expression and activity of Farnesyl Diphosphate Synthase (FDPS) in Glioblastoma.

By

Mario Abate[1,§], Chiara Laezza[2,3,§], Simona Pisanti[1], Giovanni Torelli[4], Vincenzo Seneca[5], Giuseppe Catapano[5], Francesco Montella[1], Roberta Ranieri[1], Maria Notarnicola[6], Patrizia Gazzerro[7], Maurizio Bifulco[1,3#] and Elena Ciaglia[1,#].

[1]Department of Medicine, Surgery and Dentistry "Scuola Medica Salernitana", University of Salerno, Via Salvatore Allende, 84081 Baronissi Salerno, Italy;

[2]Institute of Endocrinology and Experimental Oncology, IEOS CNR, Via Pansini 5, 80131 Naples, Italy;

[3]Department of Molecular Medicine and Medical Biotechnology, University of Naples "Federico II", Via Pansini, 80131 Naples, Italy.

[4]Neurosurgery Unit A.O. San Giovanni di Dio e Ruggi d' Aragona - Salerno's School of Medicine Largo Città di Ippocrate, 84131 Salerno – Italy.

[5]"G.Rummo" Medical Hospital, Department of Neurosurgery, Benevento,

[6]National Institute of Gastroenterology "S. de Bellis", Research Hospital, Castellana Grotte, Bari 70013, Italy.

[7]Department of Pharmacy, University of Salerno, Via Giovanni Paolo II 132, 84084 Fisciano Salerno, Italy.

[§]These authors contributed equally to this work.

[#]M.B. and E.C. are considered co-last authors.

Running Title: FDPS as a metabolic marker in glioblastoma

[*] **Correspondence:**

Maurizio Bifulco, Department of Medicine, Surgery and Dentistry "Scuola Medica Salernitana, University of Salerno, Via Salvatore Allende, 84081 Baronissi Salerno, Italy.

Tel: +39089965217 Fax: +39089969602. E-mail: maubiful@unisa.it

Elena Ciaglia, Department of Medicine, Surgery and Dentistry "Scuola Medica Salernitana, University of Salerno, Via Salvatore Allende, 84081 Baronissi Salerno, Italy. Tel: +39089965115 Fax: +39089969602. E-mail: eciaglia@unisa.it

Intraoperative brain sample sites:

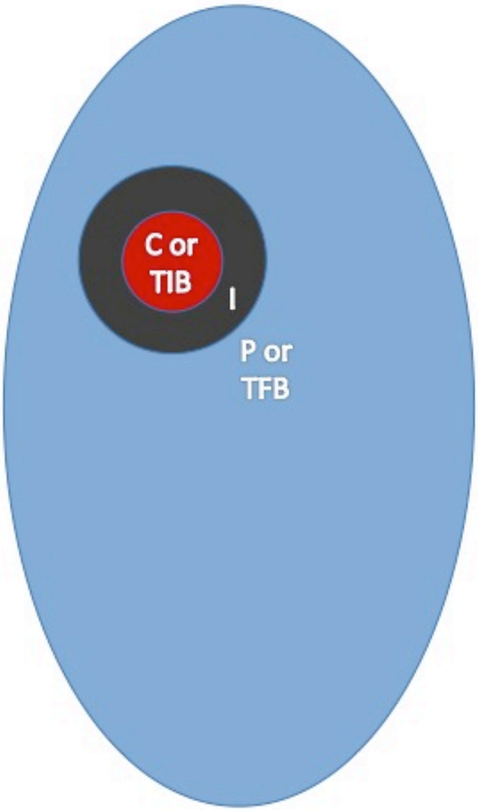

Supplementary Figure 1. ***Experimental approaches to study FDPS levels in GBM tumor brain compartments.***

As a useful strategy to overcome heterogeneity of the resected material, a potential issue in the identification of novel biomarkers, protein, enzymatic and gene expression determinations have been conducted in different intraoperative brain sample sites: inner tumor specimens (*red region*), indicated as *Central (C)* or *Tumor Infiltrated Brain (TIB)*, in the corresponding normal healthy tissue specimens (*blue region*, approximately 1 cm distant from the tumor) indicated as *Peripheral (P)* or *Tumor Free Brain (TFB)* and where possibile, in peritumoral tissue specimens (*grey region*) indicated as *Intermediate (I)* tumor fraction.

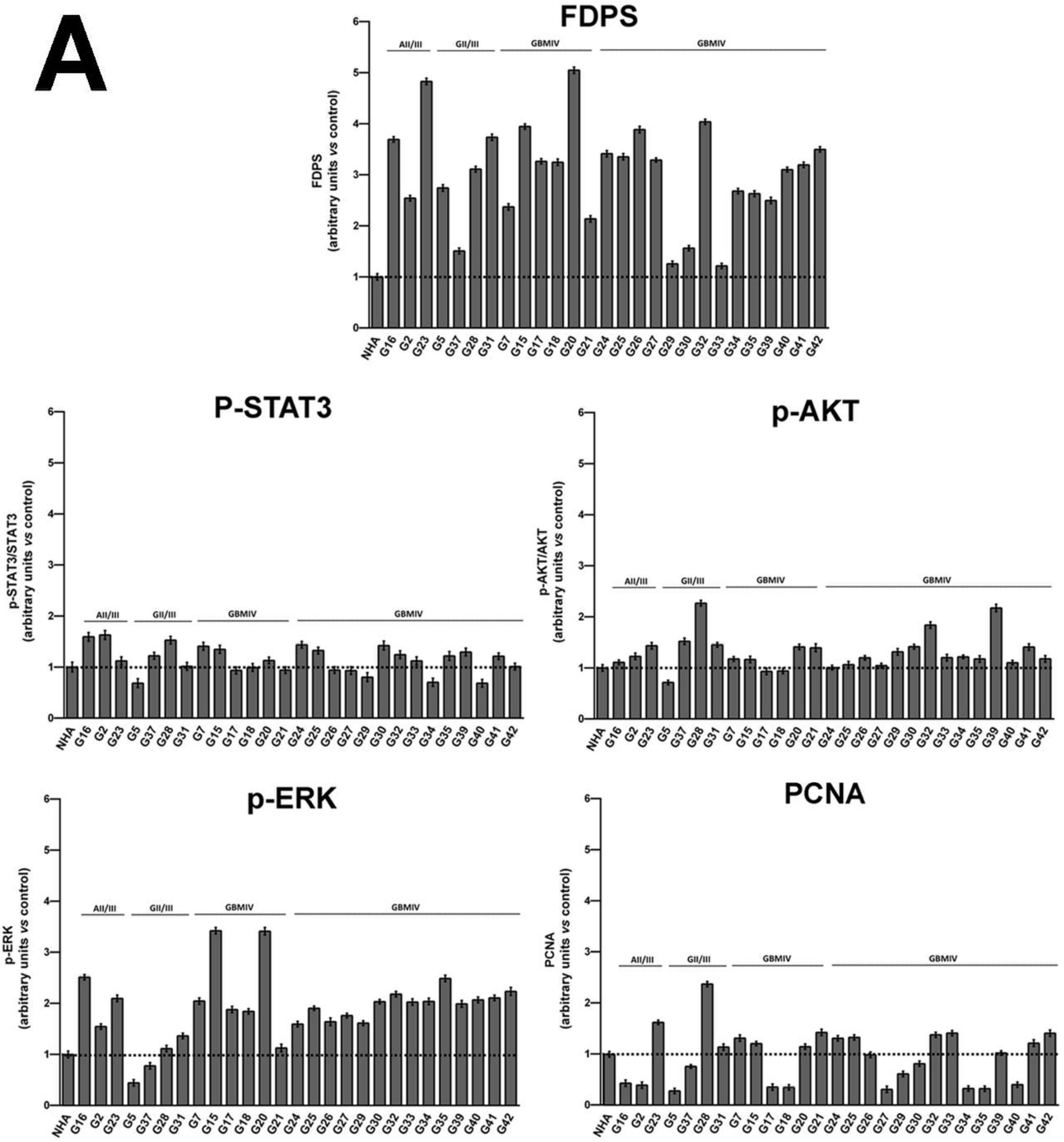

Supplementary Figure 2. ***Densitometric analysis of Figure 1B***

Histograms represent mean ± SD in densitometry units of scanned immunoblots from the 3 different experiments referred to fig. 1B.

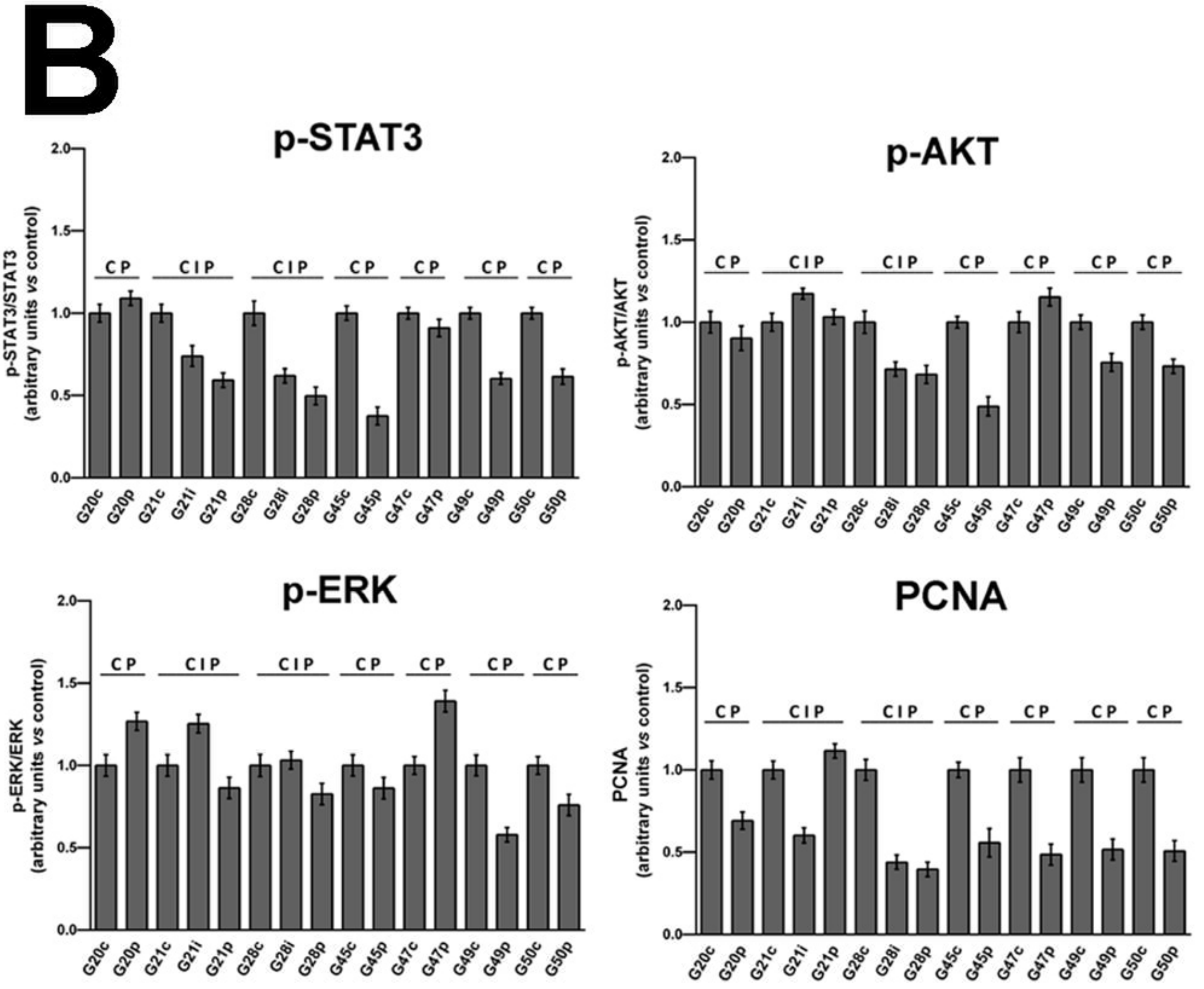

Supplementary Figure 3. ***Densitometric analysis of Figure 1C***

Histograms represent mean ± SD in densitometry units of scanned immunoblots from the 3 different experiments referred to fig. 1C.

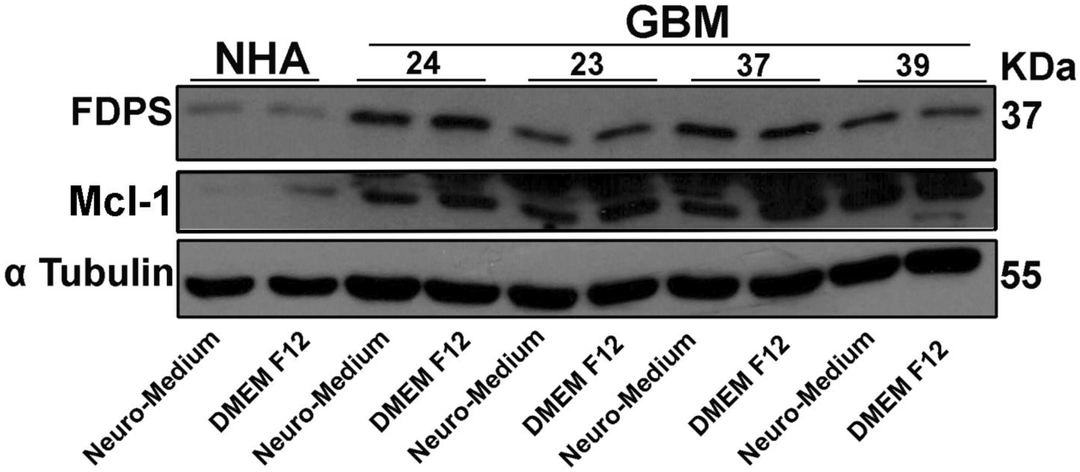

Supplementary Figure 4. ***Effect of serum based culture conditions on FDPS and Mcl-1 protein levels.***

Representative Western blot showing FDPS and Mcl-1 protein levels in both naive low passage human GBM primary cells (G24, G23, G37, G39) and in NHA as well as in primary cells and NHA cultured for two weeks in different specific culture conditions (serum free MACS® Neuro Medium *vs* conventional 15% DMEM-F12).

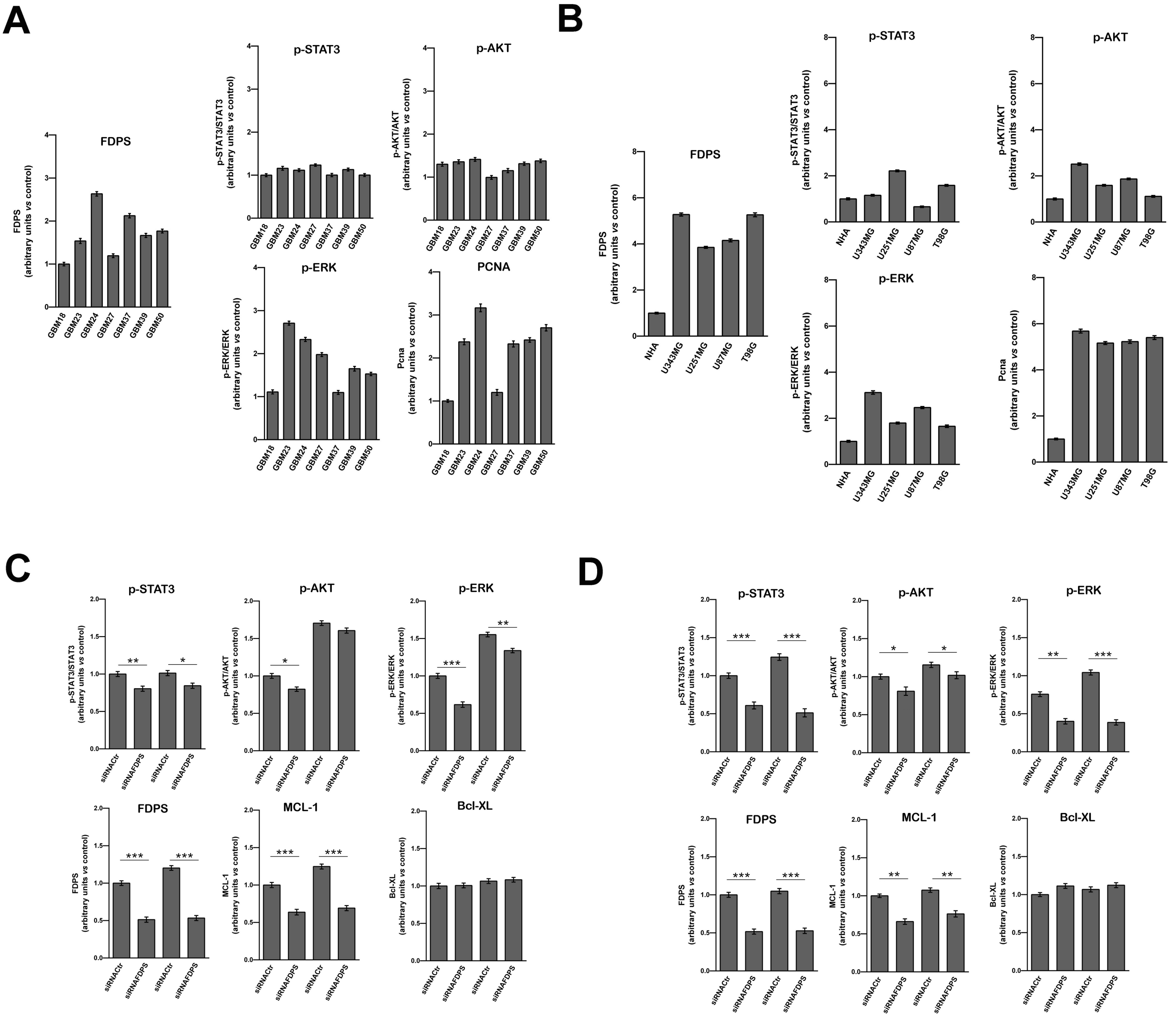

Supplementary Figure 5. ***Densitometric analysis of Figure 2***

(A) Histograms represent mean ± SD in densitometry units of scanned immunoblots from the 3 different experiments referred to fig. 2A. (B) Histograms represent mean ± SD in densitometry units of scanned immunoblots from the 3 different experiments referred to fig. 2B. (C) Histograms represent mean ± SD in densitometry units of scanned immunoblots from the 3 different experiments referred to fig. 2C. (D) Histograms represent mean ± SD in densitometry units of scanned immunoblots from the 3 different experiments referred to fig. 2F.

Clinical characteristics of the patients' cohort

Patient	Survival (months)	Hospital	Recurrence (months)	Clinical Stage	Age	Gender	Treatment post-lesion
**GBM2**	15	Benevento	9	Astro III	44	M	Chemotherapy-Radiotherapy
**GBM3**	12	Benevento	12	Astro III	63	F	STUPP Protocol
**GBM4**	18	Benevento	free	Astro III	56	M	STUPP Protocol
**GBM5**	17	Benevento	38	Glioma II	69	F	Chemotherapy
**GBM6**	8	Benevento	4	IV	73	M	STUPP Protocol
**GBM7**	16	Benevento	free	IV	49	M	Chemotherapy-Radiotherapy
**GBM8**	13	Benevento	5	Astro II	66	M	STUPP Protocol
**GBM9**	8	Benevento	6	II	58	M	STUPP Protocol
**GBM10**	10	Benevento	8	IV	55	F	STUPP Protocol
**GBM11**	13	Benevento	6	IV	63	M	STUPP Protocol
**GBM12**	11	Benevento	7	IV	72	F	STUPP Protocol
**GBM13**	17	Benevento	10	IV	48	M	STUPP Protocol
**GBM14**	12	Benevento	8	IV	54	M	STUPP Protocol
**GBM15**	9	Benevento	9	IV	73	M	STUPP Protocol
**GBM16**	9	Benevento	free	Astro II	31	M	Wait and See
**GBM17**	8	Benevento	9	IV	48	F	Chemotherapy
**GBM18**	7	Benevento	4	IV	74	M	Chemotherapy
**GBM19**	8	Benevento	5	IV	59	M	STUPP Protocol
**GBM20**	6	Benevento	free	IV	64	M	STUPP Protocol
**GBM21**	10	Benevento	4	IV	57	M	STUPP Protocol
**GBM22**	8	Benevento	5	IV	53	M	STUPP Protocol
**GBM23**	6	Benevento	free	Astroblastoma	27	M	STUPP Protocol
**GBM24**	5	Benevento	residual	IV	65	F	STUPP Protocol
**GBM25**	5	Benevento	3	IV	77	F	STUPP Protocol
**GBM26**	2	Benevento	residual	IV	66	M	STUPP Protocol
**GBM27**	2	Benevento	residual	IV	64	F	STUPP Protocol
**GBM28**	8	Benevento	9	III	52	M	STUPP Protocol
**GBM29**	8	Benevento	5	IV	43	M	Fotemustine
**GBM30**	4	Benevento	1	IV	58	F	STUPP Protocol
**GBM31**	5	Benevento	residual	Gliosarcoma	66	M	STUPP Protocol
**GBM32**	7	Benevento	7	IV	48	M	Fotemustine
**GBM33**	5	Benevento	free	IV	48	F	STUPP Protocol
**GBM34**	6	Benevento	1	IV	54	M	STUPP Protocol
**GBM35**	10	Benevento	free	IV	41	M	STUPP Protocol
**GBM36**	6	Benevento	residual	IV	69	M	STUPP Protocol
**GBM37**	alive	Salerno	26	III	70	M	STUPP Protocol
**GBM38**	18	Salerno	residual	IV	66	F	STUPP Protocol
**GBM39**	14	Benevento	10	IV	69	M	STUPP Protocol
**GBM40**	15	Salerno	residual	IV	72	M	STUPP Protocol
**GBM41**	4	Benevento	residual	IV	75	M	STUPP Protocol
**GBM42**	6	Benevento	residual	IV	73	M	STUPP Protocol
**GBM43**	9	Salerno	residual	IV	66	M	STUPP Protocol
**GBM44**	10	Salerno	residual	IV	58	F	STUPP Protocol
**GBM45**	12	Benevento	2	IV	66	M	STUPP Protocol
**GBM46**	9	Benevento	3	IV	24	M	STUPP Protocol
**GBM47**	13	Benevento	residual	IV	44	M	STUPP Protocol
**GBM48**	alive	Salerno	free	IV	49	M	STUPP Protocol
**GBM49**	11	Benevento	free	IV	50	M	STUPP Protocol
**GBM50**	10	Benevento	2	IV	47	F	STUPP Protocol

Original blots for Fig. 1B

1B

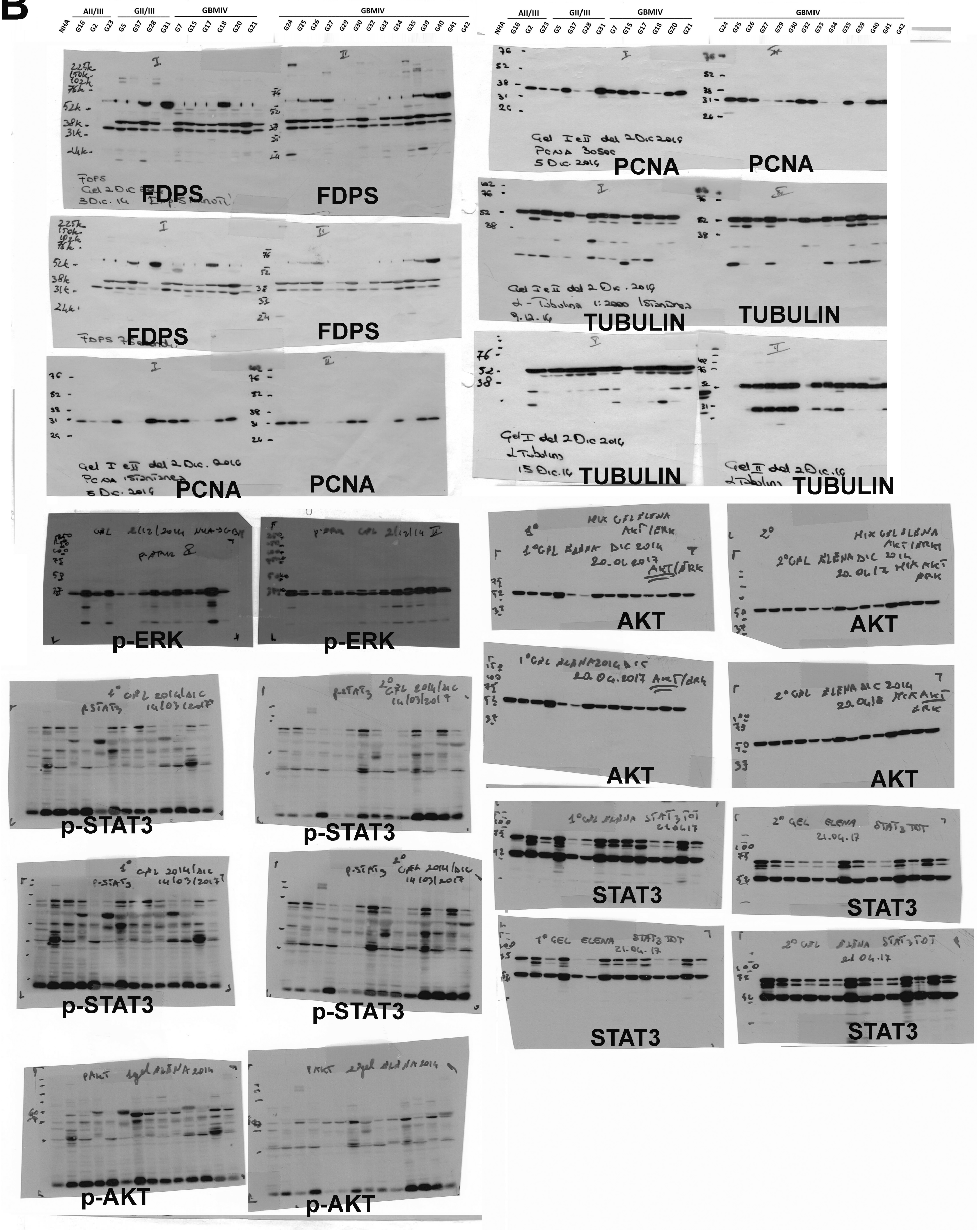

Original Blots for Fig. 1C

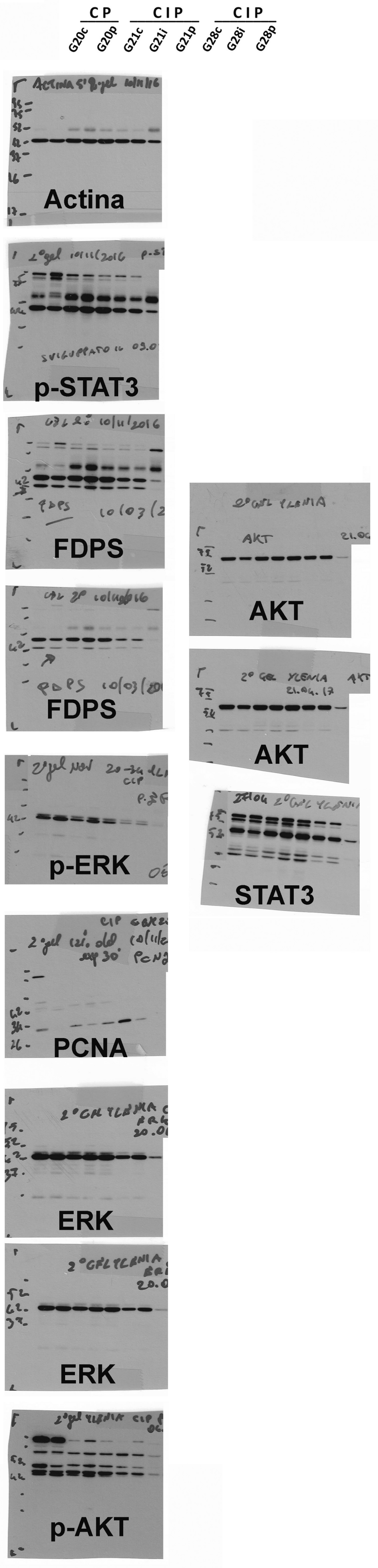

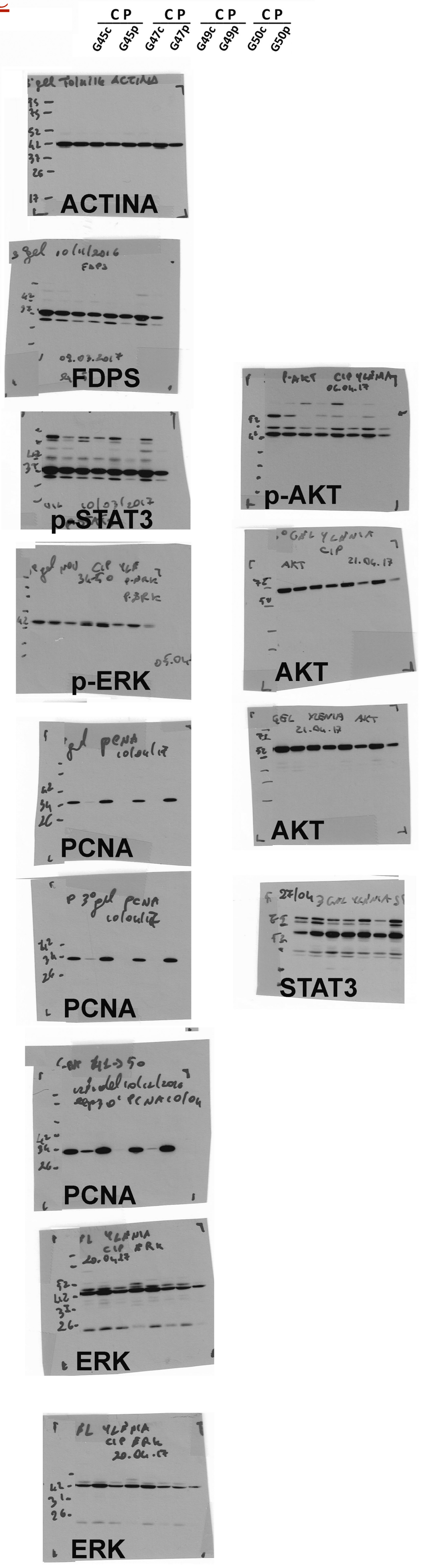

Supplement: Supplementary file 1 — Supplementary Information [file 41598_2017_14495_MOESM1_ESM.pdf]
